# Breaking the Code of Amyloid-**β** Oligomers

**DOI:** 10.1155/2013/950783

**Published:** 2013-08-31

**Authors:** Sylvain E. Lesné

**Affiliations:** ^1^Department of Neuroscience, University of Minnesota, Minneapolis, MN 55414, USA; ^2^N. Bud Grossman Center for Memory Research and Care, University of Minnesota, Minneapolis, MN 55414, USA; ^3^Institute for Translational Neuroscience Scholar, University of Minnesota, Minneapolis, MN 55414, USA

## Abstract

Departing from the original postulates that defined various neurodegenerative disorders, accumulating evidence supports a major role for soluble forms of amyloid proteins as initiator toxins in Alzheimer's disease, Parkinson's disease, frontotemporal dementias, and prion diseases. Soluble multimeric assemblies of amyloid-**β**, tau, **α**-synuclein, and the prion protein are generally englobed under the term oligomers. Due to their biophysical properties, soluble amyloid oligomers can adopt multiple conformations and sizes that potentially confer differential biological activities. Therein lies the problem: with sporadic knowledge and limited tools to identify, characterize, and study amyloid oligomers, how can we solve the enigma of their respective role(s) in the pathogenesis of neurodegenerative disorders? To further our understanding of these devastating diseases, the code of the amyloid oligomers must be broken.

## 1. Commentary

For a century, the cardinal features of Alzheimer's disease (AD), amyloid plaques and neurofibrillary tangles, were thought to underlie this chronic neurological disorder. However, based on the evidence accumulated over the past ten to fifteen years, the toxicity of these lesions has been questioned. Instead, the emerging soluble aggregation-intermediate forms of amyloid-beta (A*β*) and tau proteins, which compose plaques and tangles, are now believed to underlie the synaptic and neuronal losses observed in AD. Studies focusing on oligomeric A*β* assemblies [1–4] have paved the way for other amyloid proteins including tau [5], alpha-synuclein [6–8], and the prion protein PrP [9] in the field of neurodegenerative disorders. This principle simply revolutionized our understanding of AD, Parkinson's disease, frontotemporal dementias, and prion diseases, opening new avenues for therapeutic strategies.

In what might seem like an all rosy affair, this paradigm shift also contributed to complicating even more the putative sequence of biological events responsible for these diseases. In AD, the classical view of the amyloid hypothesis postulated that amyloid plaques are altering the physiological function of neurons, which in turn disrupts tau biology leading to the demise of the cell [10]. The modern view of the amyloid hypothesis suggests the involvement of a multitude of endogenous bioactive A*β* molecules [11] that include A*β* dimers, trimers, Aβ*56, annular protofibrils, and amyloid plaques, as opposed to a single culprit (i.e., plaques). This notion appears to be consistent with the myriad cell surface receptors and signaling pathways that have been described as specifically activated by putative endogenous soluble A*β* oligomers [11]. If this scenario was not entangled enough, numerous studies aiming at elucidating the function of oligomeric A*β* (oA*β*) use oligomeric preparations of synthetic A*β* peptides whose folding conformation and posttranslational modifications might not accurately reflect to these found in biologically relevant systems (i.e., brain, cerebrospinal fluid, blood, and primary neurons). In the end, this increased complexity of the problem coupled with a lack of adequate experimental descriptions of the oA*β* used and specific detection tools (e.g., antibodies specific to a single A*β* assembly) renders interpretation and comparison of the observed phenomena between different research groups arduous [12] and impedes on our progress to better understand the role of A*β* oligomers in AD.

A clear example of this issue plaguing our field is illustrated by the controversial debate surrounding the role of the cellular form of the prion protein (PrP^C^) in mediating the deleterious effects of oligomeric A*β*. In 2009, Lauren and colleagues reported that PrP^C^ was acting as a receptor for synthetic A*β* oligomers also called A*β*-derived diffusible ligands (ADDLs) [13, 14]. ADDLs have been characterized by denaturing electrophoresis (SDS-PAGE), transmission electron microscopy (TEM) and size-exclusion chromatography (SEC) coupled with static light scattering (SLS) [14], but each technique generated inconsistent and contradicting results. First, ADDLs ran as an undefined smear ranging from ~25 to 200 kDa using SDS-PAGE followed by western blotting with the sole 6E10 antibody detecting A*β*_1–16_. Additional bands were detected as putative monomers, trimmers, and tetramers in the ADDL preparation but since these same immunoreactive bands were also detected in freshly resuspended synthetic A*β* peptides, they are likely a result of the presence of SDS in the experimental conditions. SDS is known to artificially alter the electrophoretic migration of synthetic A*β* [15]. TEM revealed that ADDLs contained spheroidal structures of various sizes; the most abundant form appeared to correspond to 5-6 nm spheroids. It is important to note that short filamentous structures were also clearly visible possibly corresponding to protofibrils. Finally, liquid phase chromatography coupled with SLS revealed the presence of only two elution peaks under nondenaturing conditions, a broad trailing peak detected shortly after the void volume containing A*β* molecules of ~500 kDa mass and a well-defined sharp peak corresponding to monomeric A*β* peptides. The authors concluded that the preparation of ADDLs used was approximately made of an assembly composed of 50 to 100 A*β* monomers [14]. Based on the aforementioned data, it seems reasonable to conclude that these ADDLs are not stable under denaturing conditions as previously reported [16] and that the exact composition of the synthetic A*β* oligomers used remains inconclusive. Despite the apparent inconsistency of the observations characterizing the oA*β* used in this study, PrP^C^ appeared to be necessary to mediate the inhibition of long-term potentiation (LTP) induced by oA*β* [14].

As expected, this study stimulated several independent groups to reproduce these findings using various sources and preparations of A*β* [17–20]. A team led by Gianluigi Forloni first reported that PrP^C^ was not required to mediate the cognitive impairments induced by synthetic A*β* oligomers [17]. Synthetic A*β* peptides were prepared to generate ADDLs following the same groundwork established by William Klein and his colleagues at Northwestern University [13, 21]. Analyses using atomic force microscopy (AFM) and SEC defined the ADDLs and obtained and confirmed the presence of mixed structural species (i.e., spherical assemblies and protofibrils) by AFM and the presence of two elution peaks following SEC (a sharp peak close to or within the void volume and a smaller peak containing putative A*β* monomers). While these elements could suggest at first glance that the ADDLs generated at Yale and at the Mario Negri Institute are similar, it bears to mention here that the columns used in both studies greatly differed (a sequential connection of Superdex 200, Superdex 75, and Superdex peptide, 10/30, HR SEC columns for the Yale group and a single Superdex 75 column for the Italian group) raising the possibility that in fact both ADDL preparations were different.

To further demonstrate the involvement of PrP^C^ in A*β*-induced deficits, the role of PrP^C^ was examined in middle-aged APPPS1ΔE9 transgenic mice used to model Alzheimer's disease [22] expressing or deficient for the *Prnp* gene [23]. Gene deletion of *Prnp* had no apparent effect on soluble and insoluble monomeric A*β* levels as measured by western blot analyses using 6E10 despite a ~20% reduction in amyloid burden, indicating potential discrepancies in A*β* measurements and quantification. Behaviorally, ablation of *Prnp* resulted in rescuing synaptic loss, APP-induced premature mortality, and spatial learning and memory compared to APPPS1 mice [23]. Puzzlingly, CA1 LTP was not altered in APPPS1ΔE9 hippocampal slices, possibly suggesting that the endogenous A*β* species responsible for LTP inhibition are not present or that these mice might develop homeostatic compensations in response to synaptic injury induced by A*β*. In addition to the apparent inconsistency in the A*β* levels, the nature and characterization of the A*β* molecules in 12-month-old APPPS1 and APPPS1x*Prnp* ^−/−^ were not mentioned, begging the question as to whether the same A*β* species initially found to interact with PrP^C^ are the same as the hypothesized A*β* oligomers present *in vivo*.

A few months later, two independent studies published at the same time challenged the conclusions that PrP^C^ is a mediator of A*β* toxicity [18, 19]. PrP^C^ was not found to be required for A*β*-induced synaptic deficits in hippocampal slices transfected with a carboxyl terminal domain of the amyloid precursor protein APPct100 and for ADDL-induced LTP inhibition [19]. In the former paradigm, it is unknown whether oligomeric A*β* species are present in APPct100-expressing slices [19, 24], and if they were, the information pertaining to their characterization was not discussed [19]. In the second experimental condition, hippocampal slices were incubated with synthetic A*β* oligomers. Although the method used to generate ADDLs was identical to the one used by Lauren and coworkers, gene deletion of the *Prnp* gene failed to rescue the LTP inhibition induced by ADDLs. It is important to note that the characterization of the A*β* oligomers formed only included one western blot analysis with an unspecified antibody following SDS-PAGE and revealed the presence of a poorly resolved smear ranging from ~35 to ~180 kDa and monomers. In addition, the concentration at which the mixtures were used (1 *μ*M) was greater than those used by the original study (20–200 nM), possibly adding an additional confounding factor when comparing the experimental designs. Due to the absence of data describing the aggregation state of the A*β* used in these paradigms, it is difficult to conclude that the results presented invalidate the findings of the initial study by Laurén et al. [14].

The role of PrP^C^ in mediating A*β*-induced LTP deficits was investigated in hippocampal slices of 2 to 4-month-old APPPS1_L166P_ mice [25] that were genetically manipulated to express 2, 1, or 0 copies of the *Prnp* gene [18]. Contrary to earlier findings [23], LTP was impaired in an age-dependent fashion in APPPS1_L166P_ slices, but *Prnp* copy numbers did not influence the observed LTP deficits [18]. Neither full-length APP and carboxyl-terminal fragments of APP CTF*α* and CTF*β* nor soluble A*β*_42_ levels were altered by *Prnp* genotypic differences indicating that PrP^C^ does not alter APP/A*β* metabolism in this mouse model. Despite these rigorous analyses of APP derivatives, the exact nature and relative abundance of soluble A*β* assemblies present in 4-month-old APPPS1_L166P_ mice were not addressed.

In light of these disparate observations, *Nature Neuroscience* published an editorial in April 2011 entitled “State of Aggregation” which reiterated the critical need to clearly describe the initial state of the protein, its source, and its stoichiometry in order to maximize the success of independent groups that want to reproduce observed phenomena.

Shortly thereafter, Freir and colleagues confirmed that PrP^C^ is required for LTP inhibition induced by ADDLs and by protein lysates of AD brain tissue containing A*β* [26]. A major reason as to why this study stood out relies on the fact that synthetic oA*β* preparations were carefully characterized by SEC, analytical ultracentrifugation, electron microscopy, and by SDS-PAGE and that all techniques produced results that were intrinsically consistent. SEC and AUC analyses of ADDLs and biotinylated ADDLs (bADDLs) confirmed the presence of 2 peaks reminiscent of these described by Laurén et al. However, leading and trailing shoulders in the SEC elution peaks were observed suggesting the presence of species ranging from 90 to 400 kDa in the mixture, which was confirmed by AUC. Astutely, the authors also noticed that the addition of a biotin residue to A*β* artificially enriched the abundance of high-molecular weight species compared to unbiotinylated ADDLs. Using EM, both spherical and short filamentous structures were observed consistent with the profile obtained in the original study [14]. Finally, SDS-PAGE followed by 6E10 immunoblotting analyses confirmed that ADDLs are not SDS resistant and predominantly migrate as experimental artifacts as A*β* monomers, dimers, trimers, and tetramers following denaturation [15]. When this mixture was applied to hippocampal slices, LTP was inhibited in wild-type but not *Prnp*-deficient mice. Altogether, based on these biophysical observations, PrP^C^ appears to be mediating the inhibition of LTP induced by one or several unidentified synthetic A*β* oligomers. More importantly, a similar rescue of LTP inhibition was observed in *Prnp* ^−/−^ slices when Tris-buffered saline (TBS) soluble protein extracts from an AD brain were applied. Biochemical analysis of TBS fractions from AD and control brains by immunoprecipitation/western blotting revealed the presence of putative SDS-stable A*β* dimers (~7 kDa) and monomeric A*β* in AD TBS extracts, while no A*β* species were detected in control TBS lysates. It is difficult to determine whether other A*β* assemblies were present as there was substantive nonspecific background in the “no protein” condition ranging from 18 to 80 kDa and because only one antibody was used to detect A*β* (presumably 6E10).

Integrating the observations from the studies mentioned above, it seemed reasonable at the time to conclude that PrP^C^ is required for the inhibition of LTP induced by a mixture of soluble brain-derived A*β* species.

After two years of intense investigation, we still did not know the answers to the most crucial questions related to oA*β* if one aims to use this knowledge to develop diagnostic and therapeutic tools: (1) which endogenous A*β* assembly is binding to PrP^C^? (2) Where is this interaction occurring? (3) When do endogenous oA*β* engage PrP^C^? (4) How does PrP^C^ mediate the deleterious effect(s) of oA*β*? 

We sought to answer these questions combining *in vivo* experiments using human, transgenic mouse brain tissues and *in vitro* paradigms using primary neurons derived from various mouse lines [27]. To ascertain the relevance of the study, all soluble A*β* species were purified from human AD brain tissue or conditioned media of transgenic cortical neurons in liquid phase experiments (i.e., immunoaffinity capture in suspension followed by SEC) and characterized by immunoprecipitation/western blot using a panel of 4 antibodies detecting the N-terminal region (6E10), the central domain (4G8), or the C-termini of A*β* (40- and 42-end specific antibodies Mab2.1.3 and Mab13.1.1, kind gifts from Pritam Das, Mayo Jacksonville). In a reproducible fashion, we isolated endogenous A*β* monomers, dimers, trimers, Aβ*56, and protofibrillar species migrating at ~175–180 kDa in absence of any additional detectable A*β* species using our panel of A*β* antibodies. Of note, we also used the oligomer-specific antibody A11 [28] to further confirm the nature of human Aβ*56 (data not shown). Moreover, none of the purified soluble A*β* species displayed aberrant migration profiles induced by SDS-PAGE analysis (i.e., apparent monomers, dimers, trimers, and tetramers comigrating in the same lane), and all soluble A*β* captured were eluted at the predicted molecular weight during SEC, arguing against the possibility that the assemblies detected are gel artifacts. Finally, putative A*β* dimers and trimers could be found in the conditioned medium of primary mouse cortical neurons expressing the Swedish mutant form of human APP disproving that these apparent A*β* oligomers are induced by lysis or the presence of detergents. Because we thoroughly characterized and documented the initial or current state of the endogenous oA*β* present in our biological specimens, we believed we could address the who/where/when/how. Briefly, we identified that PrP^C^ formed a complex with Fyn/Caveolin-1 in AD brain tissues and that A*β* dimers were the only low-molecular oligomer that coimmunoprecipitated with this complex. Using 84 human brain specimens from the Religious Orders Study (ROS), we also demonstrated that both PrP^C^ and active Fyn (phosphorylated at Y416, pFyn) proteins were abnormally elevated in AD compared to age-matched controls and that Fyn activation was correlated to PrP^C^ expression levels [27]. We next applied a mixture of oA*β* purified from AD brain tissue containing A*β* monomers, dimers, trimers, Aβ*56, and protofibrils onto protein extracts enriched in membrane proteins derived from control subjects with no detectable A*β* species. Upon PrP^C^ pulldown, only A*β* dimers were visibly captured further validating the coimmunoprecipitation findings previously obtained using AD brain.

To determine where oA*β* could interact with PrP^C^, we performed triple-labeling immunofluorescence colocalization experiments using sections from AD and control brain and confocal imaging and image reconstruction. Soluble A*β* was identified as punctae along the neuronal processes, colocalized with PrP^C^ at dendritic spines in AD but not control brain tissue, which accounted for ~22% of oA*β* present at dendritic spines labeled with Fyn. Although the data were slightly higher (~36%), analyses performed on Tg2576 primary cortical neurons expressing A*β* monomers, dimers, and trimers generated similar results. Importantly, pFyn was also observed to colocalize with A*β* and PrP^C^ most notably at synaptic varicosities traditionally considered to reflect alterations in microtubule organization. Since tau is a microtubule-associated protein and believed to mediate A*β*-induced deficits, we analyzed tau phosphorylation status and cellular localization when PrP^C^/Fyn/oA*β* were engaged into forming an active complex. Consistent with the synaptic varicosities, tau was hyperphosphorylated at Y18, a well-known target phosphorylation site for Fyn [29], and abnormally accumulated at postsynaptic sites reminiscent of phenomena associated with synaptic dysfunction [30, 31].

It then appeared that A*β* dimers could bind to PrP^C^ engaging the activation of Fyn at dendritic spines, but knowing when this pathological event took place remained unknown. To address this question, we examined the role of aging on oA*β* in APPPS1_L166P_ mice. In 2-month-old APPPS1_L166P_, A*β* monomers and apparent A*β* trimers were readily detected albeit at low levels. In contrast, very abundant A*β* monomers and putative A*β* dimers and trimers were observed at 14 months of age. These results were consistent with earlier reports considering that A*β* dimers are associated with plaques [4, 32] and that amyloid deposition occupies ~10% of the neocortical areas at 8 months in APPPS1_L166P_ mice [25]. Further supporting the hypothesis that A*β* dimers activate the PrP^C^/Fyn complex, Fyn activation was remarkably elevated in aged APPPS1_L166P_ mice while undetectable in young animals [27]. In addition, the electrophoretic migration pattern for oA*β* did not appear to differ substantially between APPPS1_L166P_ mice expressing PrP^C^ and APPPS1_L166P_ mice deficient for *Prnp* (APPPS1_L166P_x*Prnp* ^−/−^). As predicted by our hypothesis, Fyn phosphorylation was reduced by ~50% at 14 months of age in APPPS1_L166P_x*Prnp* ^−/−^ mice suggesting that oA*β*, and presumably A*β* dimers induced the activation or PrP^C^/Fyn in aged APPPS1_L166P_ mice when amyloid burden is well established.

Finally, we sought to establish how PrP^C^ mediated the effects of oA*β*. To this end, we applied isolated A*β* monomers, dimers, trimers, Aβ*56, and protofibrils at equimolar concentrations (5 nM) onto primary cortical neurons. After 60 minutes, only A*β* dimers and trimers induced Fyn phosphorylation. Since A*β* trimers did not appear to interact with PrP^C^ based on our coimmunoprecipitations, our results pointed to A*β* dimers as the major soluble endogenous A*β* ligand for PrP^C^ *in vitro*. These findings were also in agreement with our *in vivo* data showing that *Prnp* gene deletion partly abolished Fyn activation in aged APPPS1_L166P_ mice. Tau, known to mediate A*β*-induced deficits [33], was hyperphosphorylated at Y18 in neurons treated with A*β* dimers and trimers. In aged APPPS1_L166P_ mice, removing both copies of *Prnp* diminished tau hyperphosphorylation by ~40% and missorting by ~65% compared to APPPS1_L166P_x*Prnp* ^+/−^ mice. In contrast, overexpressing PrP^C^ in APPPS1_L166P_ mice (APPPS1_L166P_xtga20) led to an ~60% increase in tau phosphorylation at Y18 and 80% in tau missorting to the postsynaptic density. Accompanying this apparent potentiation of the PrP^C^/Fyn pathway activation in old APPPS1_L166P_xtga20 mice, the expression of postsynaptic but not presynaptic proteins including the postsynaptic scaffold protein PDS-95 was reduced by ~35% adding weight to the suggestion that increasing PrP^C^ expression was potentiating A*β* dimer-induced toxicity *in vivo*.

The publication of our study was preceded by a few months by a study from the Strittmatter group whom reported that oA*β* binds to postsynaptic PrP^C^ to activate Fyn and impair neuronal function [34]. Here, synthetic oA*β* were used as previously described [14] as well as TBS-soluble extracts from individuals diagnosed with AD. Despite using 4 antibodies to identify PrP^C^-oA*β* complexes (namely, 2454, 82E1, NU-4, and AB5306) on immobilized PrP molecules, the characterization of the species detected with these antibodies in both preparations was not documented thereby hampering our ability to “put clothes on the emperor” to borrow the expression employed by Benilova et al. [12].

Instead, I am convinced that we, as a field, need to dedicate more efforts into better defining what oligomeric amyloid species are employed if we want to leapfrog towards a more comprehensive knowledge of the disease. I think we can do better than describing “a subset of peptide with deleterious actions on neurons and synapses.” 

A recent study from the Ashe and Lesné groups [32] provides support to the need of distinguishing oligomeric forms of A*β* from each other as opposed to considering them as a pool of molecules triggering the same biological effect. If correct, the findings suggest that the mixture of soluble A*β* species present in the continuum of aging AD is evolving contrasting with the determined mixture of synthetic oA*β* preparations. Specifically, Aβ*56 was most prominent in preclinical phases of AD, A*β* trimers were elevated in early symptomatic phases (i.e., mild-cognitive impairment), and A*β* dimers dominated in late symptomatic phases of AD. If longitudinal studies can confirm these changes, knowing the pathophysiological function of each A*β* oligomer in the brain could be crucial in designing therapeutic interventions. Such vision could be envisioned particularly at a time when personal medicine is emerging and when our population is aging very quickly. 

In addition, another important advance in our knowledge of AD will be to decipher where each oligomeric A*β* assembly is coming from, that is, intracellularly or extracellularly [35].

For these reasons, I believe we should encourage better characterization of the soluble forms of A*β* we use experimentally and pursue initiatives to develop new reagents specific to each oligomeric A*β* assembly (which might also allow us to identify the formation and location of A*β* oligomers *in situ*) in the hope that together we can soon break the code of the A*β* oligomer enigma.
